# Living Naked in the Cold: New Insights into Metabolic Feasibility in Primeval Cultures

**DOI:** 10.1093/biosci/biad002

**Published:** 2023-03-17

**Authors:** Richard W Hill

**Affiliations:** Department of Integrative Biology, Michigan State University in East Lansing, Michigan, United States

**Keywords:** thermoregulation, daily metabolic rate, Aboriginal peoples, Yamana (Yaghan), Bushmen (San)

## Abstract

The people of three primeval cultures lived naked or nearly naked in regions where they experienced air temperatures of ± 5 degrees Celsius during cold seasons. These were the Australian Aboriginal peoples, the Bushmen of southern Africa, and the Yamana and the Alakaluf of Tierra del Fuego. Recent meta-analyses of data on human metabolic rate and metabolic endurance enable a quantitative demonstration of feasibility: Thermoregulation at winter air temperatures while naked was feasible in the three cultures for significantly longer than 50–180 days per year (sufficient for the duration of winter). Considering the life histories of the people, their estimated, time-averaged daily (24 hours) metabolic rates in winter were 2.6 times basal—similar to the highest daily rates empirically measured in extant peoples. Although the primeval peoples’ way of life was metabolically expensive, it was as feasible as the lifestyles of peoples in today's world who live at the upper bound of the metabolically possible.

As a definitive record of one of the most  unexpected cultural phenomena in early human life, we have the words of Charles Darwin (Darwin 1839). HMS *Beagle* was anchored in the summer of 1832 at Wollaston Island, near the southern tip of South America, when Darwin encountered a canoe occupied by six Yamana (Yaghan) people. The weather that day was typical for summer in the area, with a daytime air temperature near 7 degrees Celsius (°C). In Darwin's words, “every day snow fell on the hills, and in the valleys there was rain, accompanied by sleet.” Darwin commented that the men of the region, as their sole clothing, often had “an otter skin or some small scrap about as large as a pocket handkerchief”—“barely sufficient to cover their backs as low down as their loins.” However, the people in the canoe were “quite naked” in heavy rain. Later, as Darwin and his party traveled in their vessel, “a woman, who was suckling a recently born child, came… alongside the vessel and remained there whilst the sleet fell and thawed on her naked bosom and on the skin of her naked child.” In much the same way as Yamana people approached the *Beagle* in 1832, Yamana visited the French steamship *Romanche* (figure 1) when it was in Tierra del Fuego as part of the first International Polar Year, in 1882–1883, as was described by Chapman (2010).

**Figure 1.**
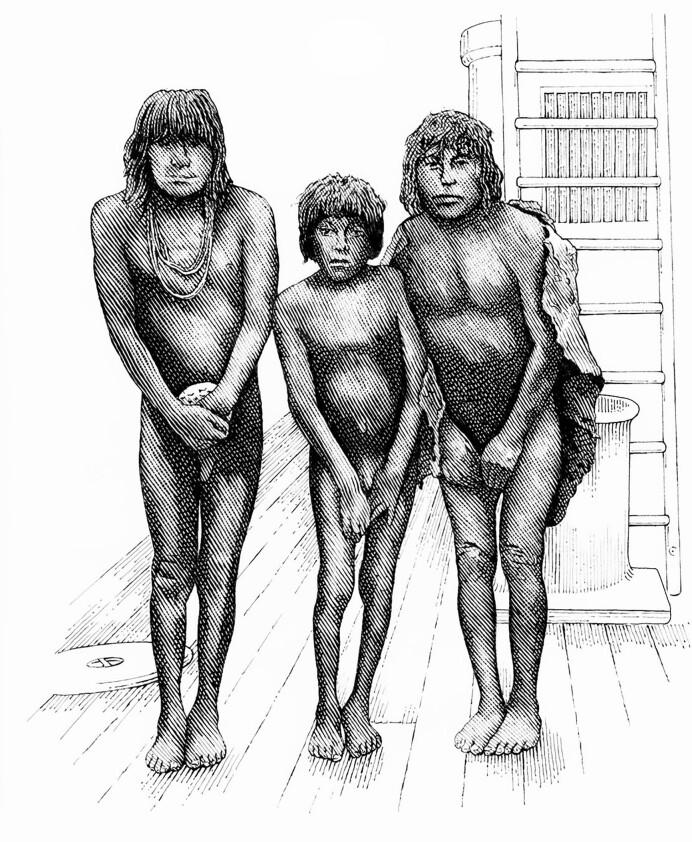
Two Yamana men and a boy who paddled out to visit the French steamship Romanche in 1882–1883, standing on the deck of the ship. Note the pelt draped over the back of the man on the right; whereas Yamana were often observed fully naked, several early observers, including Darwin, also described this type of attire (Chapman 2010). The drawing is based on a photograph that appears in Barthe and Barral (2015).

Early European explorers discovered three geographically distinct cultures in which people lived naked or nearly naked in cold climates: the Yamana and Alakaluf peoples in Tierra del Fuego, the Aboriginal peoples of Australia (see Bunda and Wilson 2012 for terminology), and the Bushman—or San—peoples of southern Africa (see Barnard 2019 for terminology). These three cultures had existed for millennia prior to discovery. The Yamana and Alakaluf were maritime peoples, and archaeological evidence indicates that they had already adopted a maritime way of life 6000 years before Europeans discovered their existence (de la Fuente et al. 2018). Australia became populated more than 40,000 years before Europeans arrived (Tobler et al. 2017), and Bushmen had lived in southern Africa for at least 11,000 years (Lee 1976, Barnard 2019).

Over the millennia prior to discovery, the peoples in the three cultures had established patterns of life by which they could succeed in some of Earth's most unforgiving environments. They therefore uniquely revealed properties and potentialities of the human species that might otherwise go unrecognized. This article arises out of respect for their achievement. Living naked in the cold has largely vanished today (at least as a freely chosen way of life) and is unlikely to be seen again in the future. It remains a thought-provoking part of human history.

For each of the three cultures, the discovery by European explorers was in many ways a tragedy. The Aboriginal peoples in Tasmania were decimated in the aftermath, for example (Jones 1971, Ryan 2012). The Yamana and Alakaluf, in fact, were driven to extinction as they were subjected to intercontinental spread of disease, armed attacks by seal hunters and gold prospectors, depletion of seals and fur seals (critical resources) by European sealing vessels, and—­ultimately—governmental assignment of the lands where they lived to owners granted property rights (Holdgate 1961, Chapman 2010).

The relatively rapid demise of all three cultures after discovery was correlated with rapid and profound transformations in the ecosystems where the peoples lived. From a conservation perspective, it is clear from recent research that, long ago, when *Homo sapiens* arrived for the first time in ecosystems previously devoid of people, significant ecosystem impacts of their arrival were common (Wilmshurst et al. 2008, Erlandson and Rick 2010, Sullivan et al. 2017). Nonetheless, in the places where the three cultures lived, ecologically complex ecosystems still existed at the time of European discovery. That is, the peoples in their indigenous state lived within complex ecosystems of which they had been a part for thousands of years. Against that background, the ecosystem changes that occurred in the first few hundred years after discovery were often massive. The amount of forested land in eastern Tasmania, for example, fell by about 60% between 1803 and 1964 in association with dramatic growth of sheep farming (Bradshaw 2012). Simultaneously, European rabbits (*Oryctolagus cuniculus*), discovered in 1827 to have escaped into the Tasmanian wild, multiplied spectacularly and sometimes ruinously for habitats (Australian government 2011, Roy-Dufresne et al. 2019).

The people in each of the three cultures were primarily or exclusively hunter-gatherers who led nomadic or seminomadic lives, moving from place to place as needed to find food or other necessities, taking all their possessions with them (see supplemental note S1). For the Yamana and Alakaluf, the principal sources of food were the seacoast and ocean. Often termed “canoe people,” they traveled in sophisticated canoes from place to place, collecting foods by wading, diving, and fishing; their diet included mussels, limpets, fish, seaweeds, otters, seals, and occasional whales (Chapman 2010). The Bushmen of southern Africa and the Aboriginal peoples of Australia had diverse diets, including plants (fruits, seeds, roots, etc.), insects, honey, lizards, fish, birds, bird eggs, and mammals of all sizes (Tindale and Lindsay 1963, Mulvaney and Golson 1971, Tanaka 1976, Yellen and Lee 1976, Lee 1979).

Why did the three cultures adopt a naked or nearly naked existence? Unfortunately, in all three cases, we have no commentary from the people who actually lived that way. Therefore, the hypotheses available are largely inventions of outsiders, who—among other concerns—have lacked firsthand knowledge of the peoples’ perceptions of spiritual life and heritage. The Yamana and Alakaluf are often posited to have avoided clothing because of the exigencies of gathering food from the sea (Chapman 2010). Early observers provided direct confirmation that the people were in and out of their canoes frequently throughout each day as they collected food (Chapman 2010), and they lived in a cloudy, rainy climate where there could be no assurance that sun and wind would dry water-soaked pelts. For the Australian Aboriginal peoples and Bushmen, the rigors of nomadism might well have been paramount in their adopting a naked or nearly naked existence. When food or drinking water ran out in locales where they lived, families faced an urgent necessity: They needed to set off on sometimes uncertain and long overland journeys to find suitable new locales. Under these circumstances, the weight and volume of possessions became obstacles to survival because the people (who typically lacked beasts of burden) had to carry all their possessions as they walked (Bleek 1928, Yellen 1976). Young children also had to be carried, diminishing the capacity to carry possessions (Lee 1979). Sometimes, loaded down, the people needed to walk in deep sand (e.g., parts of the Kalahari; Wyndham et al. 1964a) or over rough, or steep, terrain. All things considered, clothing might have seemed an unimportant hindrance to be avoided.

## A New Perspective on the Feasibility of Living Naked in the Cold

My central objective in this article is to use new research insights to advance understanding of the thermal physiology of people in the three cultures. Specifically, I develop an entirely new perspective on the basis of application and integration of two recent research advances: first, a quantitative definition of the relationship between metabolic intensity and ambient temperature in naked people (based on the meta-analysis of Hill et al. 2013 and updated in the present article) and, second, a greatly improved quantitative understanding of human metabolic endurance (e.g., Thurber et al. 2019). I develop my analysis in three steps focused on the air temperatures likely experienced by people in the three cultures, the metabolic intensities required for naked people to thermoregulate at such air temperatures, and the relationship between human metabolic endurance and metabolic intensity.

A point to stress is that I carried out step 1, the analysis of air temperatures, entirely prior to any engagement with steps 2 or 3. By doing so, because the outcomes of steps 2 and 3 are determined by formula, I ensured that my conclusions are as free as possible of distortions from a posteriori reasoning.

### Step 1: air temperatures likely experienced

Previous discussions of the three cultures have typically provided only incidental information on the temperatures they experienced. My first step, therefore, is to focus on air temperature (*T*_a_) and to define the air temperatures (*T*_a_s) to which people in the three cultures were exposed (later, I will discuss the full range of challenging conditions faced).

To obtain data on *T*_a_s, I have used records maintained by government agencies or reported in peer-reviewed literature. Moreover, I have preferentially used data from years prior to 1960, to avoid undue influence of recent anthropogenic warming. I have needed to be opportunistic because formal records of *T*_a_s decades ago can border on nonexistent in the parts of the world where the three cultures lived.

Focusing first on Tierra del Fuego, Lamb (1972), in his monumental treatise on climate, published a summary of data for 1888–1950 recorded near sea level by the meteorological station at Punta Arenas, Chile. During winter (June–September), the average *T*_a_ was 2.9°C. Moreover, the average of the monthly lowest *T*_a_s was –9.9°C, suggesting that subzero *T*_a_s near freezing were fairly common. In 1960, Hammel (1960) published statistics for five preceding years. These data were gathered at a meteorological station at sea level on Wellington Island, in the geographical range of the Alakaluf people. In the winter months, the average daily maximum and minimum *T*_a_s were 6.4°C, and 0.8°C, respectively. Chapman (2010) quoted observations from the early nineteenth century that snow usually did not lie on the ground for more than 2–3 days, and the bays never froze over.

Focusing next on Australia, I have acquired data on *T*_a_s in the first half of the twentieth century for two pertinent locations by using the databases of the Australian Government Bureau of Meteorology. One location is in the Northern Territory, where a physiological study was carried out by Scholander and colleagues (1958a) et al. (1958a) on moderate-altitude desert dwellers at Areyonga (altitude: 670 meters [m]). Data are available for the period of 1942–1951 at a nearby town at similar altitude (Alice Springs; 545 m). In winter (again, June–September), the average daily maximum and minimum *T*_a_s there were 22.0°C and 6.2°C, respectively. Tasmania is of particular interest because it is the most southern part of the country. At a lowland site (Bushy Park; altitude 27 m) near the important Aboriginal site known today as Jordan River Levee, during winter in the period of 1931–1940, the average daily maximum and minimum *T*_a_s were 12.8°C and 3.1°C, respectively. In the 1930 s, Sir CS Hicks and colleagues reported occasional *T*_a_ data during a series of physiological studies at several locations in mainland Australia. In one study carried out in August in the same general location as Scholander and colleagues (1958a), 17 measures of early morning *T*_a_ were in the range of 5.0°C–14.6°C, with half between 5.0°C and 7.8°C (Hicks et al. 1934). In another study in August at a different location, half the early morning *T*_a_s reported were 0.0°C–6.6°C (Hicks and Matters 1933). In a third study at a still different location, *T*_a_s as low as –4.0°C were reported on “frosty nights” (Hicks and O'Connor 1938).

Focusing finally on the principal home of the Bushmen, the Kalahari desert, a plateau region where the altitude is typically 800–1200 m above sea level, almost no data from prior to 1960 have been published. However, Mphale and colleagues (2018) et al. (2018) thoroughly analyzed the interannual rate of change of wintertime daily minimum *T*_a_ between 1961 and 2010, and the average rate of change was about 0.10°C per decade (see also van Regenmortel 1995). Therefore, for our present purposes, it seems straightforward to use the *T*_a_ data reported by Andringa (1984) for eight locations over the years 1958–1980. The average daily minimum *T*_a_ in June–August ranged from 2.4°C (at Tshabong in the south) to 7.9°C (at Maun in the north), with a grand average of 5.4°C; the monthly minimum *T*_a_s were typically from –3.9°C to –7.5°C. Yellon and Lee (1976) commented that, in the northern Kalahari (which is warmer than the southern; Mphale et al. 2018) during the months of June–August, the people could expect *T*_a_s below 5°C during about 60 nights per year. Van Regenmortel (1995), using data from the 1960 s to 1980 s, reported a relatively consistent relationship in the Kalahari between average winter *T*_a_ and number of days with early morning ground frost. Chilly regions in the Kalahari had about 60–70 mornings with ground frost in a year, and the median for all regions was about 30 days with ground frost per year.

Reviewing these data—and considering the opportunistic nature and sparseness of the information available—it seems true to say that cold-season *T*_a_ regimes were similar for the three cultures. Winter *T*_a_s were not consistently below freezing, but they were also not consistently moderate or balmy, as they might have been for naked people in tropical cultures. Instead, winter *T*_a_s ranged from cold, above-freezing levels to modestly below-freezing levels, with occasional dips to temperatures approaching –10°C. When I first carried out my analysis, I needed to specify a quantitative *T*_a_ range to continue, and I did this prior to engaging with steps 2 or 3, as I noted earlier. In winter, I concluded, the people probably regularly experienced *T*_a_s between positive 5°C–6°C and negative 3°C–5°C.

### Step 2: metabolic intensities required to thermoregulate

Surprisingly few direct, empirical studies have been carried out on the relationship between metabolic intensity and *T*_a_ in unclothed people. Nonetheless, some reports on men (but not women or children) have been published. Investigations of the energetics of mammalian thermoregulation are usually conducted in still air in a uniform thermal environment, and these are the environmental conditions that prevailed during the studies on naked people. In a uniform thermal environment, all surrounding surfaces have surface temperatures approximately equal to air ­temperature, *T*_a_.

Throughout this article, I express measured metabolic rates as ratios of basal metabolic rates (BMR), and I term these ratios *BMR-normalized metabolic rates*. They are also sometimes called *physical activity levels* in the literature (e.g., Dugas et al. 2011). Shetty (2005) provided an informative discussion of the utility of such ratios. The principal reason I use BMR-normalized metabolic rates is that all the original investigators who studied the metabolism–*T*_a_ relationship in unclothed people reported their results in BMR-normalized form. Such values also can be directly integrated with data on metabolic endurance in step 3.

**Figure 2.**
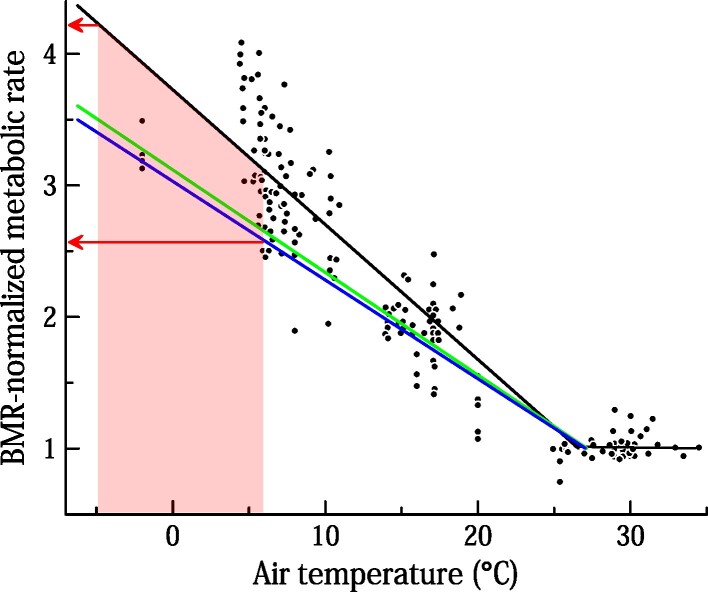
Metabolic rate, expressed as a ratio of BMR, as a function of T_a_ in essentially naked, adult men studied in still air in uniform thermal environments. The upper, black regression line and plotted symbols are from Hill and colleagues (2013). The lower two regression lines are from Ward and colleagues (1960) and refer to Bushmen (upper, green line) and South Africans of European descent (lower, blue line). The shaded zone encompasses data for T_a_s between –5°C and + 6°C. The arrows bracket metabolic rates required for thermoregulation at such T_a_s. The men in Hill and colleagues (2013) wore only skimpy shorts, and sometimes (when using a pedal ergometer) socks or shoes; those in Ward and colleagues (1960) wore only a genital cover of animal hide (Bushmen) or shorts (Europeans). Data from Hill and colleagues (2013) are presented in this figure as simple BMR-normalized ratios rather than as estimated metabolic rates as in the original report.

In 2013, my colleagues and I (Hill et al. 2013) completed a meta-analysis of all the data of which we were aware on metabolic rate as a function of *T*_a_ in naked people. The data (183 measures of metabolic rate at defined *T*_a_s) were drawn from four research reports (Erikson et al. 1956, Scholander et al. 1957, Scholander et al. 1958b; Wilkerson et al. 1972), in all of which the subjects were Northern Hemisphere adults (see supplemental note S2). The data and regression in Hill and colleagues (2013) are replotted (in black) in figure 2.

In intervening years, I have become aware of an additional study of the metabolism–*T*_a_ relationship in naked people. This study (Ward et al. 1960) is of particular note because it included subjects drawn from two populations not previously investigated: Bushmen and individuals of European descent living in southern Africa. Ward and colleagues (1960) reported their results as statistical regressions; see the blue and green lines in figure 2. The Bushmen regression was confirmed 4 years later by the same research team in a second study, carried out on a different group of Bushmen living in a different locale, using identical methods (Wyndham et al. 1964a). Moreover, the team also reported results on Bantu males (Wyndham et al. 1964ab), who were closely similar.

Taken together, all the studies that have been conducted on metabolic intensity as a function of *T*_a_ in naked people point to a relatively well defined, consistent relationship (figure 2). Of the three cultures that historically lived naked in the cold, only the Bushmen have been studied directly in this regard. However, the data available represent at least 10 different human populations (e.g., two populations are included in Scholander et al. 1957) and are relatively consistent. Therefore, it seems parsimonious and reasonable to assume that the Yamana, Alakaluf, and Australian Aboriginal peoples also exhibited the type of relationship seen in figure 2.

Earlier, in step 1, I concluded that the people of the three focal cultures living in their natural environments probably regularly experienced air temperatures between positive 5°C–6°C and negative 3°C–5°C during winter. In figure 2, the shaded zone demarks data for *T*_a_s in this range. In turn, the arrows identify the range of metabolic intensities required for thermoregulation at such *T*_a_s when people are in still air in a uniform thermal environment. Therefore, by use of the meta-analysis in figure 2, we can conclude that in winter when people in the three cultures were exposed to still air—away from fires, the radiant cooling effect of the nighttime sky, or other deviations from environmental uniformity—they required metabolic rates that were between 2.6 and 4.2 times BMR to thermoregulate.

### Step 3: metabolic endurance

As was recently summarized by Thurber and colleagues (2019), a regular relationship exists in humans between an individual's metabolic intensity and the length of time that intensity can be sustained. Therefore, now that the metabolic intensity required for thermoregulation in winter has been defined, we need to inquire whether that intensity can be sustained for the duration of the winter season.

In a groundbreaking paper, Peterson and colleagues (1990) et al. (1990) helped initiate the modern study of metabolic endurance, and an understanding of their worldview in that research is critical for present purposes. Investigators wanted to know how high the rate of metabolism can be when people must sustain an elevated metabolic rate for various lengths of time. As one example, investigators asked, “What metabolic intensity can people sustain for 22 continuous days?” A study of a 22-day Tour de France bicycle race provided an answer (Westerterp et al. 1986). During the 22 days, the cyclists traveled about 3800 kilometers and went up and down 34 mountains (Peterson et al. 1990). Each night, the cyclists took a break, during which they slept and ate. Over the entire 22 days—­taking account of both the time spent peddling and the time spent off the road—the cyclists, because they were competing to win, were highly motivated to maximize their time-averaged rate of energy expenditure, measured using the doubly labeled water method (Westerterp et al. 1986, Schoeller 1999). Therefore, the study of the tour provided data on the maximum metabolic intensity that humans can sustain for 22 days.

The time-averaged nature of such data is important to emphasize. For studies of endurance over periods of a few hours, data are gathered on people who continuously exert themselves. For example, the metabolic intensity required by a championship marathon can obviously be maintained continuously for 2.0–2.5 hours. Therefore, investigators can determine the intensity–endurance relationship for durations of 2–2.5 hours by having subjects exercise continuously during the measurement of endurance. In contrast, people must periodically rest when endurance is measured in days instead of hours. Faced with this complexity, the standard approach taken by investigators (largely for methodological reasons) has been the “real-world” approach: Metabolic intensity is quantified in a time-averaged way that includes both periods of exertion and periods of necessary rest.

**Figure 3.**
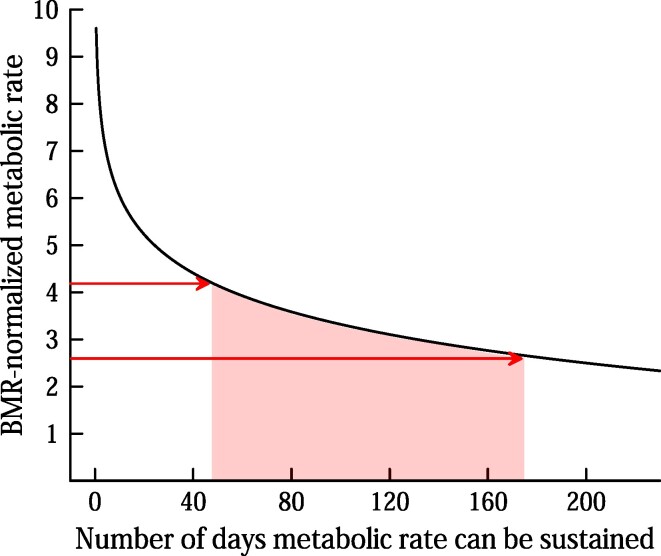
Peak BMR-normalized metabolic rate that humans can sustain for various lengths of time, as a function of length of time. This curve (black line) is based on Thurber and colleagues (2019); it is a plot of the calculated regression for their “high quality” data set (see their supplemental material, table S2). Thurber and colleagues (2019) separately analyzed data for endurance of £2.4 hours, although those data are not included in this figure. On the y-axis the arrows are drawn at values of 2.6 and 4.2: the values of BMR-normalized metabolic rate required for humans to thermoregulate while living naked in the cold, based on the analysis in figure 2. The shaded zone encompasses the lengths of time (in days) over which these metabolic intensities can be sustained.

By now, great quantities of data have accumulated on the peak metabolic rates that humans can sustain during a wide range of time periods of metabolic effort, from hours to months. By synthesizing this information, Thurber and colleagues (2019) produced the curve in figure 3, showing the functional relationship between BMR-normalized peak metabolic rate and the length of time the rate can be sustained.

Step 2 established that people in the three focal cultures needed to sustain metabolic rates 2.6–4.2 times higher than BMR to thermoregulate in winter. As is shown by the arrows and shaded area in figure 3, the relationship between metabolic intensity and endurance developed by Thurber and colleagues (2019) allows us to estimate that the people in the three cultures could sustain these metabolic rates for 50–180 days each year.

In fact, these estimates of endurance must be viewed as significant underestimates because of the time-averaged nature of the metabolic-rate measurements used by Thurber and colleagues (2019) to construct the curve in figure 3. People in all three cultures created moderated thermal microenvironments for rest and sleeping each night (see the “Environment and function during the daily nocturnal rest period” section). The metabolic rates derived from figure 2 assume immediate exposure to the specified *T*_a_s. Therefore, when we apply those metabolic rates in figure 3, we are in essence assuming that people were exposed to those *T*_a_s for the full 24 hours of each day. However, if we could have measured the people's metabolic rates in the exact same way as used in the construction of figure 3, our metabolic-rate measurements would have included time periods during each 24-hour day when the people occupied moderated nocturnal microenvironments. Time-averaged metabolic rates would have been lower than derived from figure 2, implying greater endurance. Simply because of a methodological disparity, therefore, endurance estimates derived from figure 3 by use of metabolic intensities from figure 2 are, to a significant degree, underestimates.

### Conclusion

Despite the ambiguity just noted, the analysis to this point has the virtue of parsimony: It has involved the simplest possible application of the recently established quantitative relationships in figures 2 and 3 to the interpretation of the *T*_a_ data. On the basis of this analysis, the naked people in the three cultures—when they were exposed to winter *T*_a_s—would have found thermoregulation to be quite feasible for significantly longer than 50–180 days (mean: 115 days) each year. My guess is that, intuitively, biologists today would in general view a naked existence at near-freezing temperatures to be impossible or bordering on impossible. In fact, recognizing that the June-to-September winter season nominally lasts 120 days, thermoregulation through the winter months would have been eminently feasible.

Another way to state this conclusion is to recognize that physiological capabilities place limits on cultural practices: Practices are possible only if they are physiologically permitted. The analysis just completed demonstrates that human thermoregulatory capabilities permit a lifestyle of nakedness in climates as cold as those occupied by the three cultures. Other factors, such as the exigencies of a hunter-gatherer existence, determined whether cultures actually adopted nakedness (see supplemental note S3).

In addition to figures 2 and 3, there is another branch of contemporary research that provides invaluable insight for understanding the three cultures—namely, the comparative study of daily (24-hour) metabolic rates in extant human populations. The application of this branch of research to the three cultures is notably less parsimonious than the approach adopted so far. As I will show in the “Comparative daily energetics” section, however, it strongly bolsters the argument for thermoregulatory feasibility.

## Environment and Function during the Active Hours of each Day, Including the Question of Clothing

Because of decimation or severe cultural disruption, the three cultures had largely vanished before the invention of any of the tools that biologists now use for the direct study of people in motion during their active hours of each day. No data from telemetry, dataloggers, remote sensing, or the doubly labeled water method exist for the three cultures. Therefore, descriptions of daily life in early times by explorers and anthropologists are invaluable, although such descriptions never prove sufficient to provide fully confident answers for questions in thermal biology. Few (less than five) ecophysiological studies have been conducted on daytime questions (those studies were used in constructing figure 2; e.g., Ward et al. 1960).

### Nakedness and clothing during the active hours of the day

For understanding nakedness and clothing, it is important to acknowledge two obstacles that exist. First, the people in the three cultures did not keep pertinent written or artistic records. Second, the Europeans who made first contact often urged the use of clothing to cover up nakedness, potentially requiring that clothing be worn in their presence (Dornan 1925) and initiating changes in clothing traditions.

**Figure 4.**
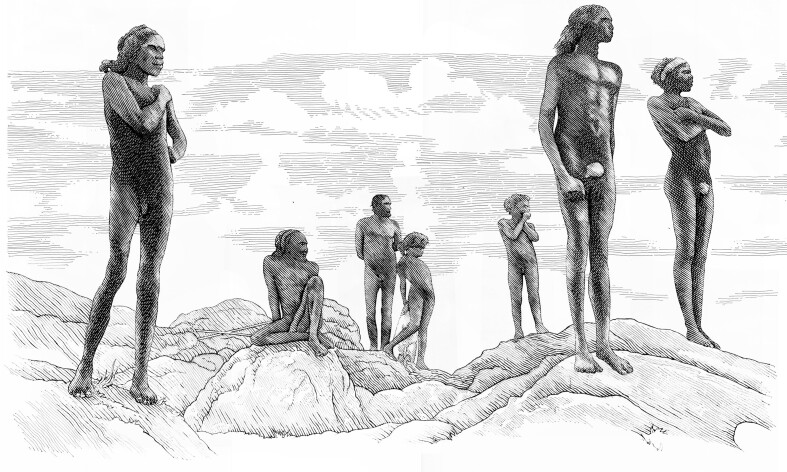
Australian Aboriginal people watching the approach of strangers in the Musgrave Ranges, Australia, probably in the early 1940 s. The people were living at Ernabella Mission, where they were encouraged to live in their traditional way, including remaining naked if they wished (Kerin 2006). The two men at the right were wearing codpieces of unknown construction. The drawing is based on a photograph that appears in Butler and Dean (2013).

Without doubt, people in all three cultures were often entirely naked as they went about their daily lives. For example, populations of Australian Aboriginals in which the people were entirely naked were described already in the 1770 s and 1780 s by reliable observers: Captain James Cook, the eminent scientist Joseph Banks (traveling with Cook), and Captain William Bligh on the *Bounty* (Gilligan, 2007, 2019). Gilligan (2007) has carried out an exceptionally thorough study of the available evidence on the Australian Aboriginals in the years before European colonization, and he concludes that “total absence of clothing was usually the case” (figure 4).

**Figure 5.**
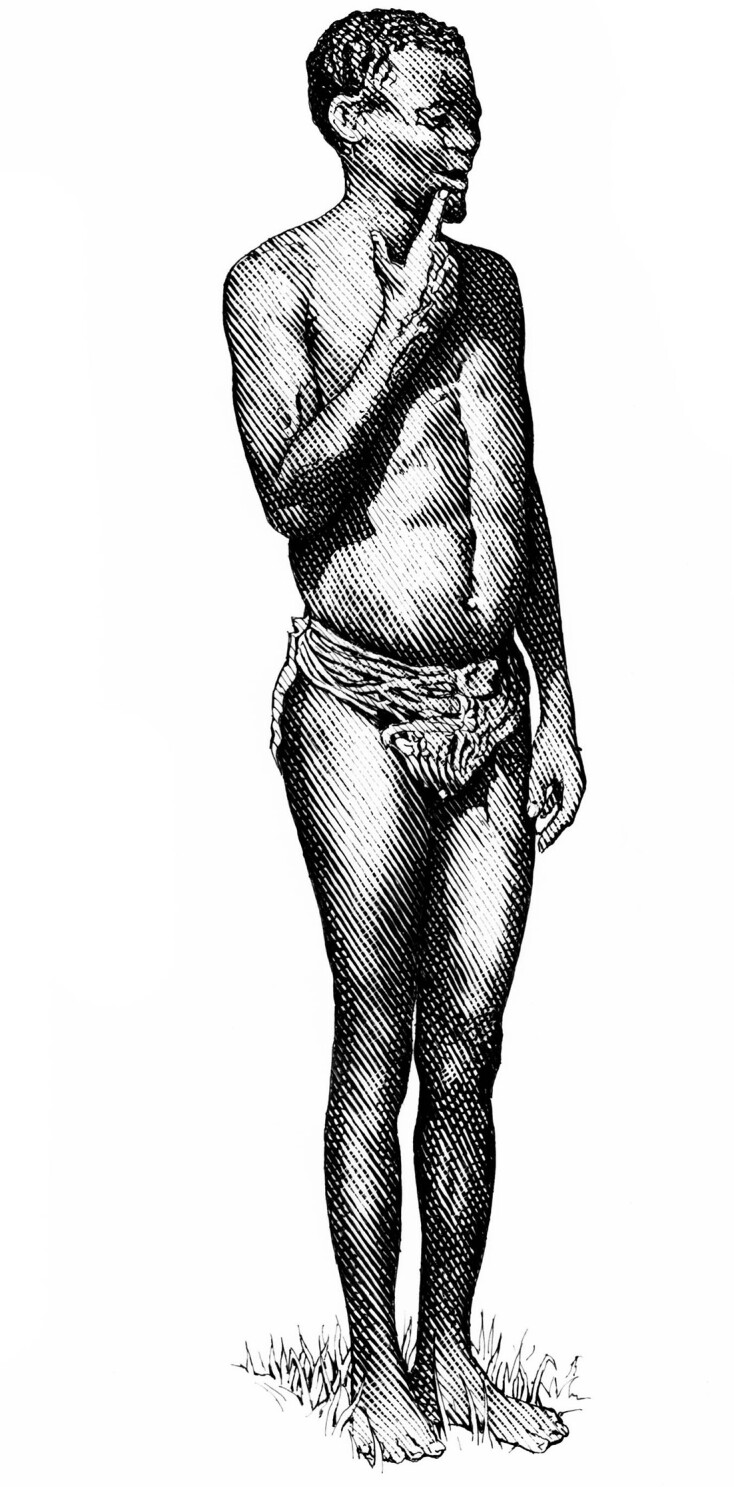
A Bushman man living in the Kalahari desert in the early 1960 s, seen in this figure in self-selected everyday attire. This man volunteered to participate in the research conducted by Hammel and colleagues (1963), and the drawing is based on a photograph in the research report.

As regards our understanding of thermal energetics in the three cultures, certain types of garments that were occasionally worn would have made no difference. Measurements on modern clothing (e.g., McCullough et al. 1985) demonstrate, for example, that a person wearing only briefs or the equivalent is virtually identical to a truly naked person in terms of heat exchange with the environment (men's knitted briefs provide 0.07 clo of body insulation, a very modest amount, recognizing that 1.0 clo is required for thermal comfort simply at room temperature, *T*_a_ ≈ 21°C; McCullough et al. 1985). Therefore, the people in the three cultures who wore only codpieces, genitalia-covering patches of animal skin, loincloths, small aprons, or other such genitalia coverings were effectively “living naked in the cold.” Possibly, the Bushman in figure 5 would have been truly naked if he had lived centuries earlier. But for understanding thermal energetics at the level at which we are capable of understanding it, the potential contrast between his state then and his state when photographed is a distinction without a difference. The entire quantitative analysis presented in the preceding “new perspective” section of the present article remains the same.

Chapman (2010), in her book-length treatise on the history of the Yamana, often recorded observations of their nakedness or attire: Certainly, the Yamana were observed naked or wearing only genitalia coverings many times over many years by visitors who encountered them living their daily lives. Similarly, Lothrop (1928) recorded Yamana use of genitalia coverings but also commented that “individuals of both sexes and all ages often went entirely naked.” We also have the words of Charles Darwin (1839) quoted previously that he saw adult Yamana both entirely naked and wearing “some small scrap.”

In regards the Bushmen, one of the earliest commentaries was that of Dunn (1931), a geologist who arrived in South Africa in 1871, when (he stated) he was able to see “the last remnant” of the precolonial Bushman culture. From direct observations and reports he received from other Europeans, Dunn concluded that the Bushmen “wore no clothes,” other than, sometimes, animal-skin genitalia coverings. Similar observations were made by Bleek (1928) and by Dornan (1925), who reported that the Bushmen were often almost naked when on their own, away from places where the onward march of European culture required them to be clothed. By 1976, when Lee and DeVore (1976) published their important book, the impacts of the modern world on Bushman culture were extensive. Nonetheless, I took advantage of the fact that the book includes many photographs of Bushmen living their daily lives. Perusing the photographs, I counted about 40 adults whom I could see well enough to be sure of what they were wearing; two-thirds were wearing no more than shorts or the equivalent. Although Viestad (2018) stated that the nakedness of Bushmen was a myth, her analysis is not physiologically relevant because she defined “dress” to include any body modification or supplement; therefore, people with merely tattoos, body paint, ­jewelry, or other ornaments were “dressed” in her vocabulary.

Individuals in all three cultures were observed on occasion to wear clothing more elaborate than genitalia coverings, although, typically when such observations were made, other individuals—living in the same general place and time—were naked or essentially naked. In regards the nature of the more elaborate garments, Ian Gilligan (2007) has provided as perfect a description as could be imagined. Speaking of the Australian Aboriginal peoples, he stated that such garments, which were made of animal skins, “were of a single layer, draped variety, hung loosely from the shoulders,” generally in the form of “capes or cloaks” (Gilligan 2007). This description is as apt for the other two cultures as for the Australian Aboriginal peoples (see supplemental note S4).

The Yamana were often observed wearing capes of otter skin, fur-seal skin, or the like. Already in the early 1820 s, the Scottish sealer James Weddell sketched these capes. His drawings of a man and woman (see figure 2.3 in Chapman 2010) depict capes too limited in size to cover the front of their bodies; the top of each cape is fastened about the neck, but each side of the cape hangs mostly over the person's back, so much so that the woman's breasts and her body below are fully exposed. Bleek (1928) reported that Bushmen were occasionally seen wearing a game hide draped from their shoulders. Among the Australian Aboriginals, Gilligan (2007, 2019) has carried out exhaustive studies of the incidence of capes and cloaks, including a map (Gilligan 2019) of the geographical distribution of particular clothing types.

When worn, capes or other draped garments—unlike mere genitalia coverings—could have substantially increased body insulation. However, the augmentation of insulation depended on how the garments were worn, their size, and the wind—factors for which we have no systematic information. Motionless air is the key to effective thermal insulation: Clothing exerts its insulating effect by creating a layer of still or relatively still air around the body (Burton and Edholm 1955, Hill et al. 2022). Therefore, when wind can shift clothing around or get under it, the insulatory value of the clothing is fundamentally degraded or negated. Because the pelts that people in the three cultures occasionally wore were draped rather than cinched around the body, wind could readily get under them. All things considered, the insulation provided by capes and cloaks—on average, in actual use—seems impossible to estimate with any reasonable level of confidence. In fact, taking a hard-headed look, it seems to me that we must wonder whether the skimpy capes worn at times, for example, by the Yamana as Darwin and Weddell reported (e.g., see the man on the right in figure 1) were worn primarily for thermal insulation; they could not have provided a great deal of insulation relative to the needs of the body as a whole and possibly were worn principally for another purpose. Chapman (2010) seemed to be expressing similar misgivings when she quoted the opinion of the eminent anthropologist Robert Lowie, who wrote that the Yamana did “not expect to be kept warm” by their skin capes.

### Fire

On the basis of the anthropological literature, it is clear that the people of all three cultures were proficient fire starters (see supplemental note S5; Bleek 1928, Lothrup 1928, Tindale and Lindsay 1963, Lee 1979, Chapman 2010). Therefore, as people moved about during the active hours of each day, fires were an ever-ready option for ameliorating the stresses of cold environments.

The canoe people, the Yamana and Alakaluf, made unique use of fire during the active hours of the day because they carried a fire with them during their daily travels as they searched for food from the sea. They installed a thick layer of soil in the center of their canoe and built a fire on it (Lothrup 1928, Chapman 2010). In principle, the Australian Aboriginal peoples and Bushmen could also have made extensive use of fire during the daily active period in cold weather, because they could have built fires wherever they stopped to rest. “Certainly,” Scholander commented, the desert peoples consistently had “ready access to dry wood of the most superb burning quality” (Scholander et al. 1958a).

Comparing the three cultures, whereas the canoe peoples, with their portable fires, might seem to be have been, by far, in the best position to be warmed by fire all day long, that would be a narrow view of their existence. With the sea as their principal source of food, they often got out of their canoes into frigid water to collect food items, even sometimes by diving (Chapman 2010). By comparison with the circumstances of the other two peoples, the transportable fires of the canoe people may merely have counterbalanced the fact that they were repeatedly getting wet.

## Environment and Function during the Daily Nocturnal Rest Period

A point worth stressing from the start of this part is that, to an extraordinary extent, the overnight rest period has been the focus of direct ecophysiological research on the three cultures. There are fewer than 20 published papers that report direct ecophysiological observations (all dating from the mid-1960 s or earlier), and most concern the rest period. This point should not be forgotten when quoting the results of the studies but often does seem to be forgotten (see supplemental note S6).

### Microenvironment during the rest period

People in all three cultures created moderated thermal microenvironments for rest and sleeping each night. In terms of thermal physics, these environments were often complex. In all three cultures, the people made extensive use of fire at night, giving rise to point sources of intense thermal radiation. In the deserts and semideserts occupied by some groups of Bushmen and Australian Aboriginal peoples, the often-clear nighttime sky could act as a significant radiant heat sink. Wind could be slight or blow vigorously. By the late 1960 s, Porter and Gates (1969) and others were starting to create computational methods sufficiently sophisticated to calculate “effective temperatures” incorporating the effects of all modes of heat transfer. But those methods were never applied in direct studies of the three cultures, and the full range of data needed to apply them was not recorded. Therefore, we can only describe the microenvironments qualitatively.

The overnight arrangements of the Australian Aboriginal peoples were particularly well described—complete with photographs—by Hicks and Matters (1933), Hicks and colleagues (1934), and Scholander and colleagues (1958a). For sleeping, the people built multiple relatively small, spaced-out fires on the ground and lay down directly on the ground naked between the fires, often quite close to the flames or embers. In addition, as they needed, the people created windbreaks with piles of brush. Scholander and colleagues (1958a) found that even they, themselves, could stay warm naked with this arrangement when the fires were burning bright and the air was still. As the fires ebbed, or if a wind came up, however, the Europeans became strikingly uncomfortable while the Aboriginal people slept. Among the Aboriginal people, someone would arise to add fuel to the fires 3–10 times in a night (Scholander et al. 1958a). The people made no provision to evade radiant heat loss to the clear nighttime sky.

Norman Tindale made over 20 long-term study trips to a variety of remote regions in Australia in the first half of the twentieth century. In his book (Tindale and Lindsay 1963), he stated simply that sleeping occurred in the way just described, indicating that the arrangement was common and widespread. The one exception Tindale noted was that huts with bark roofs and a fire inside were constructed by at least some groups in wet weather. Ellis (1972) and Ryan (2012), speaking of Tasmania, also describe bark-roofed lean-tos and huts. Where caves and caverns were available, the Aboriginal people used them at times (Gilligan, 2007, 2019).

For the Yamana and Alakaluf in Tierra del Fuego (Hammel 1960), precipitation occurred often throughout the year (more than 250 centimeters per year at Wellington Island), and winds were often strong. Correlated with these potential stresses, the people did not sleep out in the open like the Australians typically did. Instead, they constructed dwellings with at least partial roofs, inside of which fires were maintained; some of these dwellings were of a size for just a single family, whereas others could accommodate 20–30 people with multiple fires (Chapman 2010). In either case, each night, the people—adults and children—were able to cluster together with their dogs inside a fire-warmed hut to sleep, covered by any fur-seal skins or other animal pelts they might have (Chapman 2010). The huts typically consisted of a frame of branches covered with thatching of grasses and rushes or covered with animal skins (see the photo in Lothrop 1928, Holdgate 1961). An indisputable thermoregulatory advantage of the canoes of the canoe people was that the canoes enabled transport of household hides (hut roofs or blankets) when the people changed locations in their nomadic way of life. Hides, especially large ones such as fur-seal hides, are bulky and heavy. When the Australian Aboriginal peoples or Bushmen moved from one locale to another, they could take with them only the material possessions that the members of the family could carry as they walked (Bleek 1928).

Similar to the canoe people, the Bushmen also built temporary huts or other protective structures, as well as being noted for occupying natural caves or retreats in rock formations at times (Dornan 1925). Bleek (1928) and Yellon (1976) described the construction of huts, which were built on a dome-shaped frame constructed of branches and thatched with grass (for photos, see Dornan 1925, Bleek 1928, and Jones and Doke 1937). The walls served as a windbreak, and the top acted as a barrier to radiant heat loss to the clear, desert nighttime sky. A fire was positioned in front of a hut, and in winter, a second fire was made each night just inside the hut entrance (Bleek 1928, Barnard 2019).

### Physiology during the rest period

Physiological information of good technical quality for its time was gathered on people of all three cultures between 1930 and 1964. As was stressed earlier, this information pertained mostly to sleeping individuals.

Probably the most famous body of research was carried out under the direction of PF Scholander and HT Hammel, who visited Alakalufs, Australian Aboriginal people, and Bushmen, carrying out a standardized research program on all three groups (Scholander et al. 1958a, Hammel 1960, Hammel et al. 1963; summarized by Hammel 1964). The standardized program included monitoring oxygen consumption and skin temperatures all night while each subject slept individually and unclothed in a relatively standardized wool sleeping bag. Hammel quoted an average clo value of 2.8 for these bags in his summary report (Hammel 1964), signifying insulation approximately equivalent to wearing a modern outfit of all-wool winter sportswear (Hill et al. 2013). The use of sleeping bags could be criticized, because it introduced an arbitrary element into the conditions of measurement, making the data difficult to apply to any other circumstance; moreover, sleeping bags were utterly foreign to the natural ways of life of the subject peoples (see supplemental note S7). The sample sizes were small, the subjects varied in their responses, and at times, the analysis of the data was clearly post hoc.

For today, I think there are just two major conclusions of note to be drawn from the Scholander–Hammel studies. First, any physiological differences that might have existed among the peoples of the three cultures—or between them and people of European descent studied similarly—were quantitative, not qualitative. In particular, metabolic intensity was fundamentally similar in all three cultures and in the Europeans (see also the following studies in addition to the Scholander–Hammel studies: Ward et al. 1960, Hicks 1964, Wyndham et al. 1964a). Second, the Australian Aboriginal people and possibly the Bushmen tended to differ from Europeans in permitting greater cooling of peripheral tissues (e.g., feet; see the Scholander–Hammel studies, Morrison 1965). Unfortunately, this latter finding is sometimes quoted out of context, suggesting that the Aboriginal people exhibited distinctive peripheral cooling all the time. The actual data pertain only to the hours of day associated with sleep. Moreover, data gathered by other researchers do not always closely agree (e.g., Goldby et al. 1938). Of even more concern, Wyndham and colleagues (1964a) argued that the focus on sleeping (and the sleeping-bag method) may have introduced artifacts into the comparison of Bushmen with Europeans (because, whereas the Bushmen slept soundly, the Europeans did not); if this is true, the same concern might similarly apply to the comparison of Australian Aboriginal peoples with Europeans.

### Conclusion

I hypothesize that people in the three cultures were often approximately thermoneutral during the rest period of each day. To clarify terminology, I note that for an individual human (as is also often true for other mammals), metabolic rate is approximately at its lowest level when the individual is at rest and free of environmental heat or cold stress (Hill et al. 2022). The individual is then said to be in a state of *thermoneutrality*, and his or her metabolic rate is approximately equal to the basal metabolic rate, BMR.

As we have seen, the people of all three cultures marshaled numerous cultural defenses (e.g., fires, huts, windbreaks) during the rest period each day. An important point is that these cultural defenses were under the peoples’ control: In the coldest weather, they could build bigger fires, sleep closer to the fires, add fuel to the fires more often, huddle more closely together, thatch their hut more tightly to block wind, and so forth. By amplifying their cultural defenses as needed, it seems likely that the people in the three cultures were typically thermoneutral while resting at night.

Scholander and colleagues (1958a) in Australia and Wyndham and Morrison (1958) in the Kalahari recorded physiological evidence of thermoneutrality. In studies carried out without sleeping bags, Scholander and colleagues (1958a) measured the oxygen consumption of naked Aboriginal subjects throughout the night on nights when *T*_a_ fell to within a few degrees of 0°C as the subjects slept under the clear sky on the ground between fires, with bushes to the windward as a windbreak, the entire arrangement having been set up by the Australian Aboriginal people themselves. The naked individuals lying on the ground in pairs, back to back, slept soundly and maintained a metabolic rate near BMR all night (see supplemental note S8). Halfway around the world, on a milder night (*T*_a_ = 14°C–17°C), Wyndham and Morrison (1958) measured essentially thermoneutral temperatures under the leather cloak used as a blanket by a Bushman sleeping near a fire.

## Comparative Daily Energetics: Living Naked in the Cold as Simply One Version of a High-metabolic-intensity Life

Earlier, in the “new perspective” section, I established the feasibility of naked life in cold climates by use of a parsimonious analysis in which, with little further elaboration, I examined the implications of linking two already documented functional relationships: the relationship between *T*_a_ and metabolic intensity in naked people (figure 2), and the relationship between metabolic intensity and metabolic endurance (figure 3). Now that I have explored anthropological knowledge of the peoples’ daily lives, the stage is set to undertake a second metabolic analysis, less parsimonious than the first but more powerful in the insights it provides.

My goal is to interpret the three cultures in the context of the modern comparative study of human daily energetics (“daily” referring to the 24-hour day). In recent decades, as was summarized by Dugas and colleagues (2011) and others, thousands of measurements have been made of the time-averaged daily metabolic rates of people in diverse extant cultures as the people go about their day-to-day lives. Most of these measurements have been obtained using the doubly labeled water method (Schoeller 1999, Shetty 2005). What if we could go back in time and use the doubly labeled water method to measure the daily metabolic rates of the people in the three cultures living in their natural winter environments? By doing that, we would have measures that are directly comparable to measures made today on presently living people whose lives we can describe in detail.

As I emphasized earlier, when the doubly labeled water method is used to measure a person's daily metabolic rate, the person's metabolic intensity during all the hours of the 24-hour day is effectively quantified in a time-averaged way that includes periods of both exertion and rest. Therefore, for estimating the measure that would be obtained for the people of an extinct culture, we can divide each day into active and resting periods, estimate the lengths of the two periods, estimate the metabolic rate in each, and calculate a weighted average, weighted by the duration of each period. In the present analysis, I focus on winter and do the calculations using BMR-normalized metabolic rates.

I assume that, in the three cultures, the resting and active periods of each day were 8 and 16 hours long. I assume that during the resting period, the metabolic rate was approximately the same as BMR, as is justified in the “Environment and function during the daily nocturnal rest period” section. Regarding the active period, I calculated in step 2 of the “new perspective” section (see figure 2) that, for naked people directly exposed to the winter macroenvironment without wind or fire, the BMR-normalized metabolic rate averaged 3.4 (i.e., the average of the calculated extremes, 2.6 and 4.2). In the present analysis, I use the value 3.4, keeping in mind that it could be an overestimate because extensive use of fire during the active hours of the day could have lowered the value significantly, and similarly, use of capes or cloaks might have lowered it. Altogether, I estimate that in the three cultures, the BMR-normalized daily metabolic rate measured in winter by the doubly labeled water method would have been 2.6 ((3.4 × 16 + 1.0 × 8) ÷ 24; see supplemental note S9). With this value in hand, the comparative method can be used to gain insight into its meaning.

Dugas and colleagues (2011) found that if all published measures of BMR-normalized daily metabolic rate for people in today's world are compiled, the grand mean value is around 1.7–1.8. More pertinent for our purposes are the upper extreme values. Anthropologists and sociologists have established that, in some extant cultures, people lead lives of such sustained, exceptional intensity that they maintain daily metabolic rates far higher than average, at least during one or two seasons of the year and sometimes for the entire year. From studies of such people, we have direct measurements of the highest daily metabolic rates that people freely choose to sustain in their day-to-day lives over long periods of time.

Thurber and colleagues (2019) highlight one example: male farmers in Gambia (see the supplemental material for Thurber et al. 2019). During the rainy-season harvest months, these farmers work intensely in their fields for 120 days of each year cultivating groundnuts, rice, millet, and sorghum by manual labor for local consumption. The farmers are motivated to maximize their crop by the fact that food stores built up during the harvest season have a history of being nearly depleted before the next harvest season begins (Minghelli et al. 1990). Heini and colleagues (1996) et al. (1996) employed the doubly labeled water method and a second method to estimate the farmers’ BMR-normalized daily metabolic rates as the men engaged each day in their “extremely high level of physical activity,” obtaining an average value of 2.4.

Table 1 summarizes three additional examples in today's world in which people engage in freely chosen lifestyles that demand exceptionally high daily metabolic rates. In these cases, BMR-normalized values of 2.2–2.3 have been measured.

**Table 1.** Particularly high daily costs of life measured by the doubly labeled water method in peoples alive today.

		Average BMR-normalized daily (24 hours) metabolic rate	
People studied and source	Lifestyle	Men	Women
Shuar peoples in Ecuadorean Amazonia (Christopher et al. 2019)	Subsistence farming, gathering, and hunting	2.3	1.8
Herders in Finland (Ocobock et al. 2021)	Multimonth roundup of reindeer herds	2.3	1.9
Agropastoralists in Bolivia (Kashiwazaki et al. 2009)	Herding and subsistence farming, measured in period of high farming activity	2.2	2.3

Abbreviation: BMR, basal metabolic rate.

A key document for the interpretation of variance in daily metabolic rate is the report of the Joint FAO/WHO/UNU Expert Consultation (FAO 2001), which is the latest iteration of a classic series of publications on human daily energy requirements. Focusing on BMR-normalized daily metabolic rates “that can be sustained for a long period of time by free-living adult populations,” the report categorizes values of 2.0–2.4 as reflecting “vigorous or vigorously active” lifestyles, with 2.4 being approximately a maximum. Shetty (2005), summarizing the views of an international group of experts, reached similar conclusions. A question that is not fully settled is the extent to which daily metabolic rates affected by high costs of physical labor have bearing for interpreting lifestyles in which costs of thermoregulation are particularly high. However, Thurber and colleagues (2019) emphasized that in their comprehensive analysis of peak sustained metabolic rates, values having widely different causes adhere to a single relationship between rate and endurance. Thurber and colleagues (2019) concluded from their empirical survey that a value of about 2.5 is likely the highest BMR-normalized daily metabolic rate that people can maintain, for any reason, for a long period of time without drawing down body energy reserves.

All things considered, I think it is true to say that my estimated value for people in winter in the three cultures, 2.6, is indistinguishable from the highest values known empirically to exist in human populations alive today. I conclude from this that, although living naked in the cold was energetically an especially demanding way of life, it was as feasible as the highly demanding lifestyles chosen by the Gambian farmers studied by Heini and colleagues (1996) and the other extant peoples listed in table 1. Living naked in the cold was simply one version of a high-metabolic-intensity life that people alive today prove is possible.

This revelation may help explain why the people in the three cultures stayed naked through hundreds of generations. From what we know, they felt no need of coverings for genital modesty and in fact had no self-conception of being “naked” (Gilligan, 2007, 2019). Therefore, their only reason to add more clothing would have been to provide increased thermal insulation, and they may have felt little need for that either (or else they saw negative effects of clothing that outweighed the positive effects of which they were aware).

An important point emphasized earlier also deserves to be recalled: The people in the three cultures had control over some of the key determinants of their daily metabolic energy costs. If BMR-normalized costs were tending to exceed the human maximum of about 2.4–2.6, the people could have made more use of fire or clothing during the active hours of each day, and they could have increased time spent in the protective microenvironments that they created for rest and sleeping.

Certainly, our knowledge of peoples’ lives in the three cultures is far too meager to address such nuances beyond listing possibilities. Feasibility may be the only question we can answer, and according to the comparative evidence available on human daily energetics, their way of life was as feasible as the lifestyles of people in today's world who live at the upper bound of the metabolically possible.

## Supplementary Material

biad002_Supplemental_File

## Author Biographical

Richard Hill (hillr@msu.edu) is a professor emeritus in the Department of Integrative Biology at Michigan State University in East Lansing, Michigan, in the United States. He is lead author of Animal Physiology, now in its fifth edition, published by Oxford University Press and Sinauer Associates.
