# Supplementary material for: Living Naked in the Cold: New Insights into Metabolic Feasibility in Primeval Cultures
Source: Bioscience. 2023 Mar 17;73(3):182–95. doi: 10.1093/biosci/biad002 (PMC10148593; doi:10.1093/biosci/biad002)
Supplement: biad002_Supplemental_File [file biad002_supplemental_file.docx]

Supplement

Living Naked in the Cold: New Insights Into Metabolic Feasibility in Primeval Cultures

Richard W Hill

Contents: Supplemental notes *N*1 – *N*9

Supplemental note *N*1. Although few long-term records of camp movements have been published, Yellon (1976) provides a detailed (perhaps particularly dramatic) record for a group of Bushmen. The group moved the location of its camp more than 40 times in a year.

Supplemental note *N*2. During the empirical studies in one of the four investigations (Wilkerson et al. 1972), the subjects simply shivered to increase their metabolic rates as needed to thermoregulate in the cold. In the other three investigations (Erikson et al. 1956; Scholander et al. 1957, 1958a), an exercise-based method was used: Each subject employed a pedal ergometer to regulate exercise thermogenesis for thermoregulation in the cold. The exercise-based method allowed the unclothed subjects to endure exposures to low *T*_a_s without the discomfort of sustained, intense shivering. *T*_a_-specific metabolic rates measured by the two approaches were similar, and Hill et al. (2013) combined the data from all four investigations to estimate the functional form of the metabolism-*T*_a_ relation.

In the study by Ward et al. (1960) described in the next paragraph in the main text, subjects shivered to increase their metabolic rates for thermoregulation.

Supplemental note *N*3. A question that inevitably arises is whether people in the three cultures were physiologically specialized. If in fact life in the three cultures was feasible with metabolic characteristics similar to those seen in general populations of people today (Figures 2 and 3), the specializations (if any) might well have been in sensory, rather than metabolic- thermoregulatory, biology. Observational evidence exists that the people in the three cultures had perceptual responses to cold very different from those of the investigators who visited them. For example, the indefatigable Per Scholander and his fellow investigators attempted to sleep naked under identical conditions as Australian Aboriginal people on cold nights. Their experience depended on the severity of cold, wind, and other factors. In one attempt, however, Scholander commented that the investigators “shivered and thrashed about all night,” and for them “very little sleep was possible,” while simultaneously the Australians slept soundly (Scholander et al. 1958b). When a breeze came up, the conditions became “almost unbearable” for the investigators while the Australians continued sleeping (see also Goldby et al. 1938). Although evidence exists that perceptual tolerance to cold can undergo relatively rapid acclimatization during an individual’s adult life (Scholander et al. 1958a, Brȕck et al. 1976; see also Young et al. 1986), studies have never been carried out on the effects of lifelong cold exposure starting at birth, nor on possible evolutionary specializations. Scholander et al. (1958b) described the “stoic indifference to pain” they perceived in the Australian Aboriginal peoples and posit that it may be relevant to understanding reactions to cold.

Supplemental note *N*4. A point worth mention is that relatively elaborate garments were not necessarily worn *in addition* to a genital covering. Quite often, when elaborate garments were worn, they were all that was worn, even if they did not obscure the genitalia (e.g., see the man on the right in Figure 1; see also Gilligan 2019).

Supplemental note *N*5. In desert regions, such as occupied by the Australian Aboriginal peoples and Bushmen, the people commonly started fires by rotating a stick of wood in a depression in wood of different hardness (Bleek 1928, Tindale and Lindsay 1963, Lee 1979). However, friction methods of this sort work best with thoroughly dry implements and were probably of very limited utility in rainy habitats. Chapman (2010) asserts that the only method that the Yamana and Alakaluf knew to start fire was the use of pyrite and a striking stone (see also Lothrup 1928).

Supplemental note *N*6. I presume that accessibility was the reason for the bias toward doing studies in the rest period. Hunter-gatherers move around a lot, and presumably the people were most reliably accessible for investigators to visit in the rest period.

Supplemental note *N*7. In their reports, Scholander and Hammel indicated that they used the sleeping bags to ensure that subjects would not experience cold stress so severe as to overwhelm their physiological defenses.

Supplemental note *N*8. Under similar conditions (Scholander et al. 1958b), Scholander himself and his fellow investigators, also naked (positioned each to himself), did not get much sleep but attributed this problem in part to the hard ground and frequent need to get up to feed the fires. Provided the wind was low, they reported being thermally comfortable all night, and they also maintained a metabolic rate near BMR all night. Scholander et al. (1958b) also quoted unpublished observations by the members of another research team of European descent who slept in the same way: “They found that they themselves could easily derive enough heat from these fires to be comfortable and even sweat, while resting naked behind the low windbreak at an air temperature of 0°C.”

Supplemental note *N*9. *BMR-normalized metabolic rate* and *physical activity level (PAL)* have the same meaning, as earlier noted (see Dugas et al. 2011).
